# Novel Semiconductor Cu(C_3_H_3_N_3_S_3_)_3_/ZnTiO_3_/TiO_2_ for the Photoinactivation of *E. coli* and *S. aureus* under Solar Light

**DOI:** 10.3390/nano13010173

**Published:** 2022-12-30

**Authors:** Ximena Jaramillo-Fierro, María Fernanda Cuenca

**Affiliations:** 1 Departamento de Química, Facultad de Ciencias Exactas y Naturales, Universidad Técnica Particular de Loja, San Cayetano Alto, Loja 1101608, Ecuador; 2 Departamento de Producción, Facultad de Ciencias Exactas y Naturales, Universidad Técnica Particular de Loja, San Cayetano Alto, Loja 1101608, Ecuador

**Keywords:** semiconductors, reactive oxygen species (ROS), bacterial photoinactivation, *Staphylococcus aureus*, *Escherichia coli*, solar light

## Abstract

The use of semiconductors for bacterial photoinactivation is a promising approach that has attracted great interest in wastewater remediation. The photoinactivator Cu-TTC/ZTO/TO was synthesized by the solvothermal method from the coordination complex Cu(C_3_H_3_N_3_S_3_)_3_ (Cu-TTC) and the hybrid semiconductor ZnTiO_3_/TiO_2_ (ZTO/TO). In this study, the effect of photocatalyst composition/concentration as well as radiation intensity on the photoinactivation of the gram-negative bacteria *Escherichia coli* and the gram-positive bacteria *Staphylococcus aureus* in aqueous solutions was investigated. The results revealed that 25 mg/mL of photoinactivator, in a Cu-TTC:ZTO/TO molar ratio of 1:2 (*w*/*w*%) presents a higher rate of bacterial photoinactivation under simulated solar light (λ = 300–800 nm) in comparison to the individual components. The evidence of this study suggests that the presence of the Cu(C_3_H_3_N_3_S_3_)_3_ coordination complex in the ZnTiO_3_/TiO_2_ hybrid semiconductor would contribute to the generation of reactive oxygen species (ROS) that are essential to initiate the bacterial photoinactivation process. Finally, the results obtained allow us to predict that the Cu-TTC/ZTO/TO photocatalyst could be used for effective bacterial inactivation of *E. coli* and *S. aureus* in aqueous systems under simulated solar light.

## 1. Introduction

Water is essential for the maintenance of plant and animal life, so the conservation of this resource must be assumed as a priority for the inhabitants of the planet [1]. Currently, as a result of human activities, there are worrying levels of various contaminants in the water, which deteriorate the quality of this natural resource and make it potentially dangerous, preventing its use and consumption [2,3]. Large amounts of effluents from various sources are usually disposed of through direct discharge; however, this method is not environmentally friendly because it adds a significant number of contaminants to the natural water resource. Additionally, some wastewater has a high nutrient composition that can provide a suitable habitat for a variety of microorganisms such as viruses, bacteria, fungi, and parasites that kill natural aquatic life. Consequently, safe disposal and proper treatment for the reuse of wastewater are presented as global sustainability strategies [4,5].

The gram-negative bacterium *Escherichia coli* is a representative foodborne pathogen as is the gram-positive bacterium *Staphylococcus aureus*. These microorganisms have been detected in surface water and wastewater and can survive on material surfaces for a long time [6]. The literature mentions several diarrheal intestinal diseases caused by pathogenic strains of *E. coli*; likewise, it is indicated that 65–75% of cases of urinary tract infections are caused by this bacterium [7,8]. Among the main bacteria that have been shown to colonize the nose is the ubiquitous bacterium *S. aureus*, which is a normal microbiota of mucous membranes and skin that can also behave as an opportunistic pathogen that causes serious diseases [9,10]. Therefore, the application of effective methods of disinfection of aqueous effluents, which guarantee clean and safe access to this vital resource, is of great importance [11].

For the efficient reduction of most microorganisms in aqueous systems, chlorination has been proposed as a conventional method since it allows for reduction of the incidence of infectious diseases, it is easy to use and it is low cost. However, despite these advantages, some health effects are mentioned, such as skin and eye irritation, as well as the generation of by-products that are toxic and modify the taste and smell of water [12]. Faced with these secondary effects and limitations, alternative methods have been proposed for wastewater treatment, including other biological, physical, and chemical processes such as ion exchange, reverse osmosis, biological denitrification, adsorption, and photocatalysis [13]. Among these methods, disinfection mediated by photocatalysts has advantages such as the use of green energy (sunlight) and the non-existent formation of toxic by-products [14].

The application of photocatalysts based on titanium oxide (TiO_2_) for the elimination of microorganisms has gained great importance in recent years [15,16]. In fact, several studies have reported the advantages of using this oxide for antimicrobial purposes due to its ability to produce reactive oxygen species (ROS) that damage the cell membrane [17,18]. In addition, TiO_2_ has other advantages such as its high photocatalytic activity, physical and chemical stability, low price, and low toxicity [19,20,21,22]. However, the disadvantage of this oxide is related to the high rate of generation and recombination of charge carriers and the band gap that only allows it to adsorb ultraviolet light which represents only 5% of sunlight spectrum [23,24,25]

Currently, due to the important evolution of photoassisted technologies for microbial disinfection, the development of electron transport materials (ITEM) [26,27,28,29] with great quantum efficiency, suitable for photochemical application under sunlight radiation (~300–800 nm), has been promoted [30]. Among the alternatives used for the development of these materials, the coupling of semiconductors, doping, and the use of sensitizers are mentioned [31]. Semiconductor coupling allows the combination of the particular characteristics of the components in an integrated semiconductor whose stability and final physicochemical properties depend on the compatibility between the individual semiconductors [32]. In a previous study, we reported that ZnTiO_3_ is suitable for the potential coupling of TiO_2_ [33] since, like other coupling semiconductors such as ZnO, Fe_2_O_3_, CuO, ZrO_2_, SiO_2_, MgO, MoO_3_, SnO_2_, CdS, ReS_2_ and WO_3_ [34,35,36,37], it allows a higher efficiency in charge separation, a long lifetime of charge carriers and a better interfacial charge transfer towards the adsorbed substances [38,39].

Doping semiconductors with metal or non-metal ions is also a common method to reduce electron–hole recombination (e^−^/h^+^) and extend the action of the photocatalyst against visible light. In relation to TiO_2_, which has a wide band gap (~3.2 eV), some effective dopants are mentioned, such as Au, Mo, Co, Rb, La, Ag, or Cu [40,41,42], which cause the band gap to decrease through the formation of defective states [43]. These states serve as active centers to trap electrons, limiting their mobility and increasing the separation of charge carriers [44,45]. The doping of TiO_2_ with Cu has been widely studied for antibacterial purposes [46,47,48], since this element has a favorable ratio of bacterial inactivation and cell damage, without causing a decrease in biocompatibility [49,50]. The integration of Cu into TiO_2_ can increase the donor density and improve charge transport through the shift towards the TiO_2_ conduction band (CB). In addition, Cu facilitates the formation of ROS on the surface of TiO_2_, generating greater reactivity and consequently greater damage to the bacterial cell membrane [51,52].

On the other hand, several studies have shown that the photochemical efficiency of TiO_2_ can be improved by the use of sensitizers [53,54,55,56], including transition metal coordination complexes, such as the Cu(C_3_H_3_N_3_S_3_)_3_ complex (Cu-TTC), which was referenced in previous studies [57]. This complex formed by Cu(I) and trithiocyanuric acid (C_3_H_3_N_3_S_3_) proved to be an effective photosensitizer in the Cu(C_3_H_3_N_3_S_3_)_3_/ZnTiO_3_/TiO_2_ (Cu/TTC/ZTO/TO) system since it allowed the insertion of e^−^ between the conduction band of the hybrid semiconductor ZnTiO_3_/TiO_2_ and the excited molecule [58]. Furthermore, the Cu-TTC complex demonstrated highly efficient charge transfer from metal to ligand and was suggested as a generator of reactive oxygen species (ROS), which are essential for microbial inactivation processes.

The Cu[C_3_H_3_N_3_S_3_]_3_ (TTC) complex presents an interesting structure consisting of three molecules of trithiocyanuric acid (C_3_H_3_N_3_S_3_) that act as ligands and coordinate with a central core of Cu(I). Trithiocyanuric acid (TTCA), also known as 2,4,6-trimercapto-1,3,5-triazine or simply trimercaptotriazine, presents a symmetric conformation characterized by the presence of three sets of σ-donor atoms (N and S), which exhibit a high capacity to stabilize transition metals in high oxidation states [57]. Furthermore, the literature reports that in many coordination complexes, trithiocyanuric acid often exists as a tautomeric structure, either as a thione or a thiol [59], as shown in Figure S1: Tautomeric structures of trithiocyanuric acid (TTCA).

Several studies have highlighted the potential of trithiocyanuric acid to coordinate with metallic elements (Ni^2+^, Cu^2+^, Zn^2+^, Cd^2+^, Hg^2+^, Ag^+^ and Tl^+^) through N and/or S atoms, or even through the SH-C=N group, forming a η^2^-fashion chelation. Consequently, due to the availability of σ donor atoms, the TTCA ligand has been able to act as a bridge in several polymeric structures reported in the literature, including the Cu-TTC complex [Cu(C_3_H_3_N_3_S_3_)_3_] [60,61,62].

On the other hand, also in a previous study, the chemical structure of the Cu-TTC/ZTO/TO compound was suggested and supported by DFT studies. In that study, the Cu-TTC/ZTO/TO compound presented the coordination sphere of the of the tautomer-thione Cu-TTC in the form of a trigonal dipyramid anchored on the surface of semiconductors. In fact, in the Cu-TTC/ZTO/TO compound, the two triazine rings acted as bidentate ligands that coordinated through their S atoms with a Ti atom on the ZnTiO_3_ and TiO_2_ surfaces (Figure S2: Anchoring modes of the Cu-TTC molecule on the surface of (a) ZnTiO_3_ and (b) TiO_2_), the coordination on the ZTO surface being more energetically stable than on the TO surface at the calculation level used.

Finally, the results of previously reported experimental and theoretical studies then allowed us to suggest the feasibility of using Cu-TTC as a possible ROS-generating photosystem, as well as a photosensitizer of the heterogeneous ZnTiO_3_/TiO_2_ photocatalyst. However, despite these interesting results, the fact that, to date, there are no studies on the bacterial photoinactivation capacity of the novel photocatalyst Cu-TTC/ZTO/TO motivated the authors to carry out this study for the first time, which is also intended to evaluate the effect of the operational parameters, concentration/composition, of the photocatalyst and intensity of the radiation in the rate of photoinactivation of the gram-negative bacteria *Escherichia coli* and the gram-positive bacteria *Staphylococcus aureus* in aqueous solutions.

## 2. Materials and Methods

### 2.1. Materials

All reagents were purchased from commercial sources and used without further purification: Trithiocyanuric acid [C_3_H_3_N_3_S_3_] (Sigma-Aldrich, St. Louis, MO, USA, 95.0%), Copper(II) perchlorate hexahydrate [Cu(ClO_4_)_2_·6H_2_O] (Sigma-Aldrich, St. Louis, MO, USA, 98.0%), N,N-Dimethylformamide [(CH_3_)_2_-N-CHO] (Fisher Scientific, Waltham, MA, USA, 99.9%), Isopropyl alcohol [C_3_H_8_O] (Sigma Aldrich, St. Louis, MO, USA, ≥99.5%), Titanium(IV) isopropoxide [Ti(OC_3_H_7_)_4_] (Sigma Aldrich, St. Louis, MO, USA, 98%), Acetic acid [CH_3_COOH] (Fluka, 99.8%), Hydrogen chloride [HCl] (Fisher Scientific, Waltham, MA, USA, 37%), Cetyl-trimethyl ammonium chloride [C_19_H_42_NCl] (Sigma Aldrich, St. Louis, MO, USA, 25%), Hydrogen peroxide [H_2_O_2_] (Sigma Aldrich, St. Louis, MO, USA, 35%), Silver nitrate [AgNO_3_] (Sigma Aldrich, St. Louis, MO, USA, >99.8%), Nitric acid [HNO_3_] (Sigma Aldrich, St. Louis, MO, USA, 69%), Zinc acetate dihydrate [Zn(CH_3_COO)_2_·2H_2_O] (ACS, St. Louis, MO, USA, ≥98%), Trypticase Soy Broth (Fisher Scientific, Waltham, MA, USA), Trypticase Soy Agar (Fisher Scientific, Waltham, MA, USA), Gibco^TM^ Gentamicin (Fisher Scientific, Waltham, MA, USA).

Samples of *Escherichia coli* (ATCC 25922) and *Staphylococcus aureus* (ATCC 25923) were obtained from the American Type Culture Collection (ATCC, Manassas, VA, USA) to test bacterial inactivation. The irradiation of the bacteria on the photocatalyst-dispersed solutions was carried out using simulated solar light by a solar box (ATLAS, SUNTEST CPS+), equipped with an air-cooled 1500 W Xenon lamp (Atlas Material Testing Technology, Mount Prospect, IL, USA). Irradiance was set to 250 W/m^2^, and wavelengths of 300–800 nm (without cut-off filter) or 400–800 nm (with 400 nm cut-off filter) were allowed to pass through. Field effect scanning electron microscopy (SEM) images of the composites were obtained on a Zeiss Gemini ULTRA plus electron microscope (Carl Zeiss AG, Oberkochen, Germany) operating at 3.0 kV. The samples for SEM measurements were dropped and dried on a piece of silicon wafer.

### 2.2. Synthesis and Characterization of ZTO/TO and Cu-TTC/ZTO/TO Nanocomposites

The ZnTiO_3_/TiO_2_ (ZTO/TO) nanocomposite was synthesized by the sol–gel method, while the complex of Cu(I) and trithiocyanuric acid (Cu-TTC) was synthesized by the sedimentation method, as we explained in previous studies [33,57]. The Cu-TTC/ZTO/TO photocatalyst consisting of the coordination complex Cu(C_3_H_3_N_3_S_3_)_3_ and the semiconductors ZnTiO_3_ y TiO_2_ [58] was synthesized by a routine solvothermal method as follows. A total of 250 mg of Cu-TTC were dispersed in with 30 mL of water and 10 mL of C_3_H_8_O. This mixture was kept under sonication at room temperature for 2 h, after which an adequate amount of the ZTO/TO hybrid semiconductor was added to obtain the composite Cu-TTC/ZTO/TO with the proportions (*w*/*w*%) Cu-TTC:ZTO/TO of 1:1 (composition P1), 1:2 (composition P2), and 1:4 (composition P3). The resulting suspensions were stirred for 24 h at room temperature to obtain Cu-TTC/ZTO/TO particles suspended in the water/C_3_H_8_O solution. Then, each solution suspension was placed in a 100 mL Teflon-lined autoclave and kept at 100 °C for 12 h. The final Cu-TTC/ZTO/TO composites were obtained by precipitation, then washed and dried at 60 °C. A color change of the samples from white to brown-yellow could be detected. For SEM measurements, the samples were dropped and dried on a piece of silicon wafer. On the other hand, the point of zero charge (PZC) of both photocatalysts was determined by the pH drift method (ΔpH = pH_f_ − pH_i_ = 0) in the range of pH 3–11 and at room temperature (22 ± 2 °C). Points of Zero Charge (PZC) are pH values at which the surface charge components become equal to zero under given conditions of temperature, applied pressure, and composition of the aqueous solution. This does not mean that the surface is discharged at pH_PZC_, but that there are equal amounts of positive (+) and negative (−) charges. In this study, the pH drift method was performed by adding identical amounts of material to a set of solutions of the same ionic strength at different pH values. In a series of 50 mL centrifuge tubes, 0.1× *g* of sample was added to 25 mL of a 0.1 M NaCl solution. The pH was adjusted with 0.1 M HCl and 0.1 M NaOH as necessary to obtain the proper pH range. The pH values of the supernatant in each tube were denoted as pH_i_. Samples were shaken for 24 h using a rotary shaker at 220 rpm. After precipitation, the pH values of the supernatant in each tube were measured and reported as pH_f_. The PZC was obtained from the graph of ΔpH (pH_f_ − pH_i_) vs. pH_i_. The assays were repeated with 0.05 and 0.01 M NaCl solutions. Each set of experiments was performed in triplicate and the mean value was recorded [63].

### 2.3. Evaluation of Bacterial Photoinactivation

To determine the antibacterial effect of photocatalyst, the time–kill test was used. According to the literature, this test is a strong tool for obtaining information about the dynamic interaction between the photocatalyst composite and the bacterial strains [64]. The time–kill test allowed us to investigate the antibacterial effect as a function of the concentration and composition of the Cu-TTC/ZTO/TO photocatalyst, as well as a function on the type of irradiation using two bacterial strains. Gram-negative *E. coli* strain (ATCC 25922) and gram-positive *S. aureus* strain (ATCC 25923) were first incubated overnight at 37 °C in Trypticase Soy Broth. For the determination of the antibacterial effect dependent on the concentration and composition of the photocatalyst, aliquots of the respective overnight cultures in NaCl/KCl (pH = 7) were placed in Petri dishes containing Cu-TTC/ZTO/TO dispersed in water at different concentrations (0.1 mg/mL, 0.5 mg/mL, 1 mg/mL, 5 mg/mL, 10 mg/mL, 25 mg/mL and 50 mg/mL). The determination of the antibacterial effect depending on the type of radiation was carried out under simulated solar light (full and with 400 nm cut-off filter) for 4–8 h. The pH measurement was performed before and after the photoreactions. The final equivalent concentration of the bacterial cultures in the Petri dishes was 5 × 10^6^ CFU/mL according to the McFarland scale. In both assays, growth controls consisting of bacterial culture of adjusted final concentration without addition of photocatalyst as well as in the absence of radiation were used. The antibiotic Gentamicin (1 mg/mL) was also used as positive control to compare the antibacterial effect of the materials synthesized under the conditions tested.

After each determination, the respective solutions were transferred to a 2 mL sterile Eppendorf tube containing 1 mL of autoclaved NaCl/KCl saline. Subsequently, these solutions were thoroughly mixed using a Vortex for 3 min. Serial dilutions were prepared in NaCl/KCl solution. An aliquot of 100 µL was pipetted onto a nutrient agar plate and then spread over the surface of the plate. Agar plates were incubated, lid down, at 37 °C for 24 h before colonies were counted [48]. Three independent assays were performed for each photocatalyst. The percentage of dead cells in relation to the number of initial live cells (CFU/mL) in each tube was then calculated by the agar plate count method. IBM SPSS Statistics 25 for Windows (Version 25.0., Released 2017, IBM Corp., Armonk, NY, USA) was used to collect the data and calculate the measures of central tendency. The results were expressed as mean values.

To verify that no regrowth of either *E. coli* and *S. aureus* occurs after the first cycle of bacterial inactivation, the bacteria–photocatalyst suspension was incubated for 24 h at 37 °C. Then, 100 µL of bacteria–photocatalyst suspension were placed in three Petri dishes to obtain replicas. The samples were incubated at 37 °C for 24 h. No bacterial regrowth was observed in these samples.

## 3. Results

### 3.1. Characterization of ZTO/TO and Cu-TTC/ZTO/TO Nanocomposites

The crystallographic phases of the photocatalysts ZTO/TO and Cu-TTC/ZTO/TO were reported in a previous study [58]. In that study, the presence of zinc titanate (ZnTiO_3_) and the anatase phase (TiO_2_) in both photocatalysts was demonstrated, as well as the presence of the Cu-TTC complex in the Cu-TTC/ZTO/TO composite. The relative amount of the ZTO and TO crystallographic phases in the ZTO/TO photocatalyst was estimated at 47% and 53%, respectively, while the relative amount of the Cu-TTC, ZTO and TO crystallographic phases in the Cu-TTC/ZTO/TO was estimated at 22%, 37%, and 41%, respectively.

Likewise, the UV–vis absorption spectra of the suspensions of the materials evaluated here were previously determined [58]. From these results, we present here the values of the molar extinction coefficients (ε) and the bandgap energy (E_g_) for ZTO/TO, Cu-TTC, and Cu-TTC/ZTO/TO. The optical band gap energy (E_g_) values were calculated by extrapolation using the (αhv)^2^ vs. hv plot and the following expression [65]:

(1) $$E_{g}=\frac{1240}{\lambda },$$

where E_g_ is the bandgap energy in electronvolts (eV) and λ represents the lower cut-off wavelength in nanometers (nm). On the other hand, the values of the molar extinction coefficients (ε) were calculated using the Lambert-Beer’s law [63]

(2) A=εCL,

where A is the absorbance at the first extension absorption peak, C is the molar concentration of the material, and L is the thickness of absorption cell usually as a constant. Table 1 shows the information regarding the maximum absorption peaks, molar extinction coefficients (ε), and bandgap energy (E_g_) for ZTO/TO, Cu-TTC, and Cu-TTC/ZTO/TO, which were evaluated at a concentration of 2.5 × 10^−5^ M.

**Figure 1:**
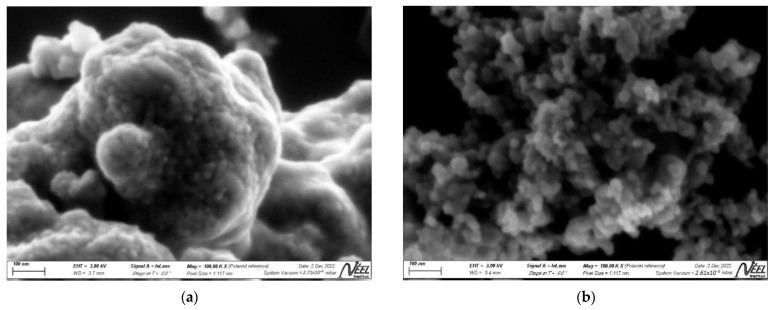
SEM images of (**a**) ZTO/TO and (**b**) Cu-TTC/ZTO/TO nanocomposites.

In this study, we also report the morphology and point of zero charge (pH_PZC_) of the photocatalysts. Figure 1 shows the SEM images of the ZnTiO_3_/TiO_2_ and Cu-ZnTiO_3_/TiO_2_ nanocomposites. The images reveal that the particles of both composites are almost spherical, highly agglomerated and have an average particle size of 25 and 35 nm for ZTO/TO and Cu-TTC/ZTO/TO, respectively.

**Figure 2:**
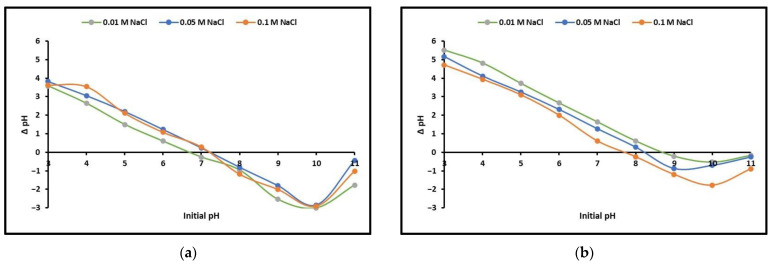
Diagrams of initial pH vs. ∆pH for determination of point of zero charge of (**a**) ZTO/TO and (**b**) Cu-TTC/ZTO/TO nanocomposites.

On the other hand, the point of zero charge was determined to be pH_PZC_ 7.0 ± 0.2 and 8.2 ± 0.2 for ZTO/TO and Cu/TTC/ZTO/TO nanocomposites, respectively. The point of zero charge was determined as the average of the plot lines at each ionic strength with ∆pH = 0, as they are shown in Figure 2. Therefore, the bacteria could have been involved in the electrostatic attraction (physisorption mechanisms) by a positive charge due to the protonation of the photocatalyst surfaces below the pH_PZC_.

### 3.2. Evaluation of Bacterial Photoinactivation as a Function of Catalyst Concentration

**Figure 3:**
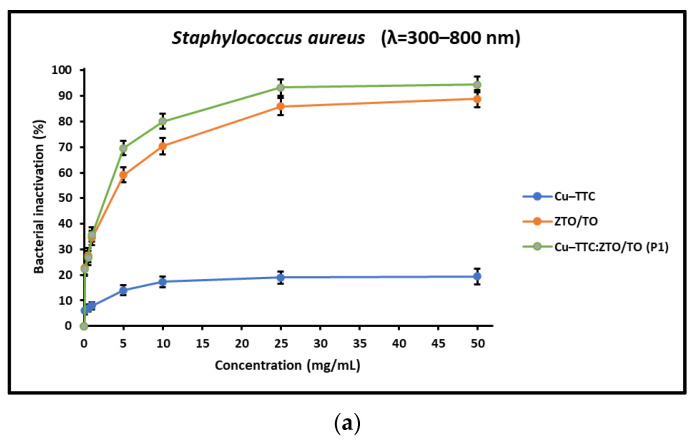

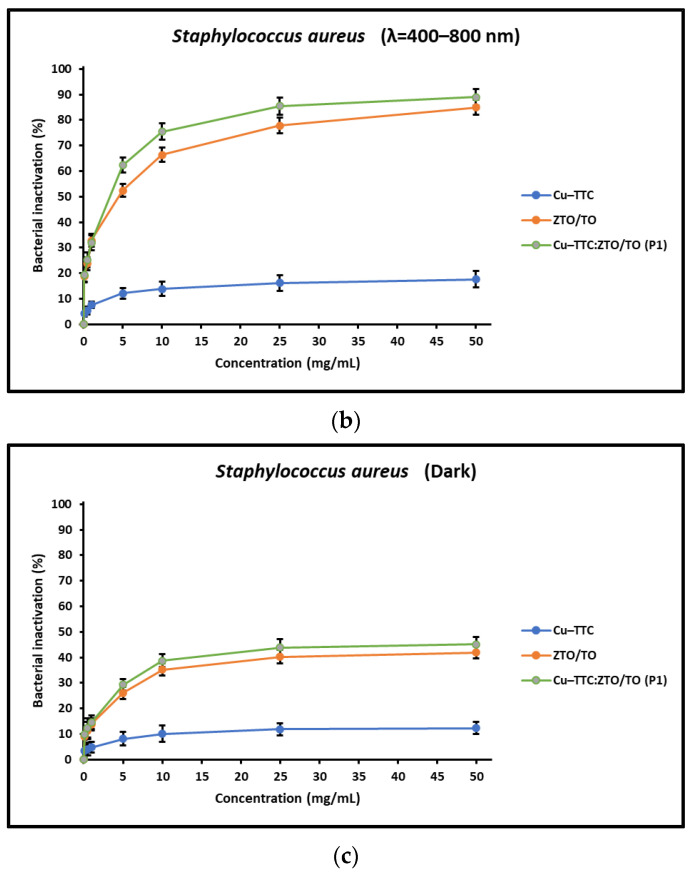
Inactivation of *S. aureus* as a function of catalyst concentration under (**a**) full simulated solar radiation, (**b**) with a 400 nm cut-off filter, and (**c**) in dark conditions.

**Figure 4:**
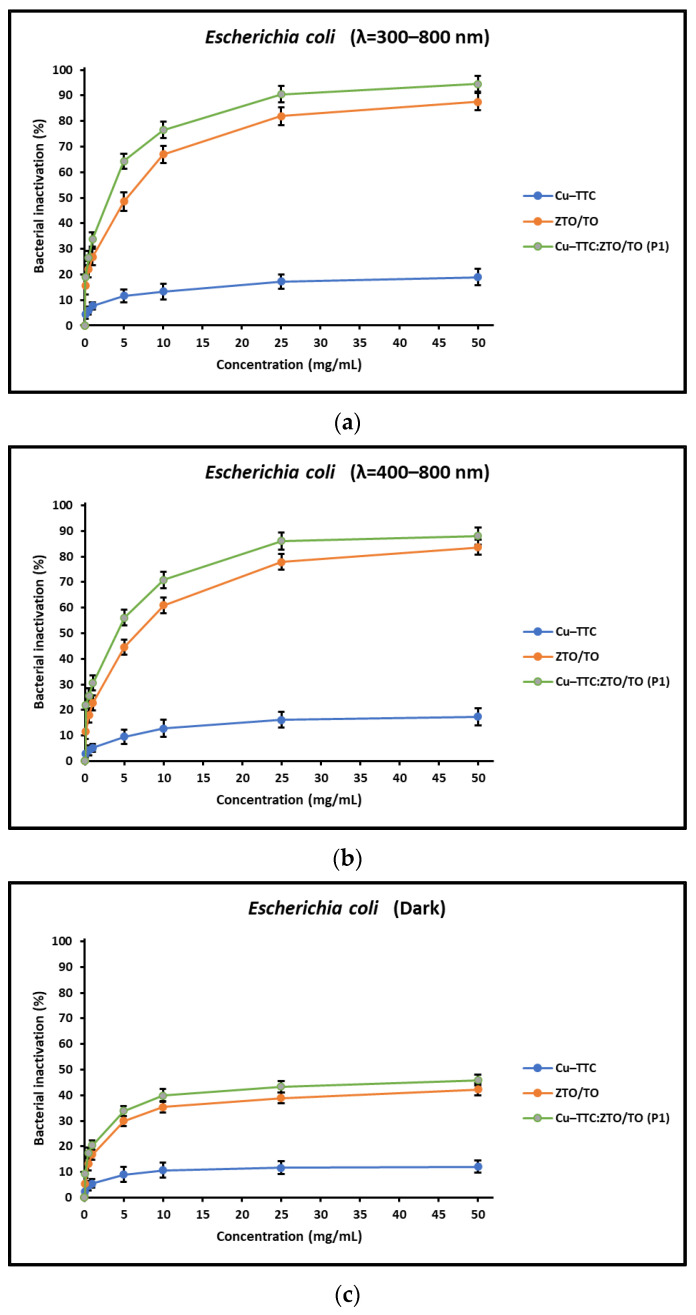
Inactivation of *E. coli* as a function of catalyst concentration under (**a**) full simulated solar radiation, (**b**) with a 400 nm cut-off filter, and (**c**) in dark conditions.

Bacterial inactivation as a function of catalyst concentration was first evaluated. For each bacterium, seven different concentrations (0.1 mg/mL, 0.5 mg/mL, 1 mg/mL, 5 mg/mL, 10 mg/mL, 25 mg/mL and 50 mg/mL) of photocatalyst were used for the photoinactivation assay under simulated solar light (full and with 400 nm cut-off filter) for about 4.5 h. Bacterial inactivation curves for *E. coli* and *S. aureus* are shown in Figure 3 and Figure 4, respectively. The percentage of bacterial inactivation for Cu-TTC/ZTO/TO in composition P1 was higher than the percentage of bacterial inactivation for ZTO/TO and Cu-TTC. Figure 3a–c show that for the photocatalyst Cu-TTC/ZTO/TO, the maximum percentages of reduction in the bacterial load of *S. aureus* were 94.4%, 88.9% and 45.1% under full simulated solar radiation, with 400 nm cut-off filter and in dark conditions, respectively. For the ZTO/TO photocatalyst, the maximum percentage of bacterial decrease were 88.9%, 84.9% and 42.0%, under full simulated solar radiation, with 400 nm cut-off filter and in dark conditions, respectively.

Likewise, Figure 4a–c show that for the photocatalyst Cu-TTC/ZTO/TO, the maximum percentages of reduction in the bacterial load of *E. coli* were 87.6%, 83.6% and 42.3% under full simulated solar radiation, with 400 nm cut-off filter, and in dark conditions, respectively. For the ZTO/TO photocatalyst, the maximum percentages of bacterial decrease were 18.9%, 17.3% and 12.1%, under full simulated solar radiation, with 400 nm cut-off filter, and in dark conditions, respectively. Finally, in this study, the Cu-TTC complex showed slight antibacterial activity for both *E. coli* and *S. aureus* under the tested conditions. According to the literature, the TTC ligand can induce antibacterial properties, not dependent on light, in transition metal coordination complexes [66].

### 3.3. Evaluation of Bacterial Photoinactivation as a Function of Catalyst Composition

**Figure 5:**
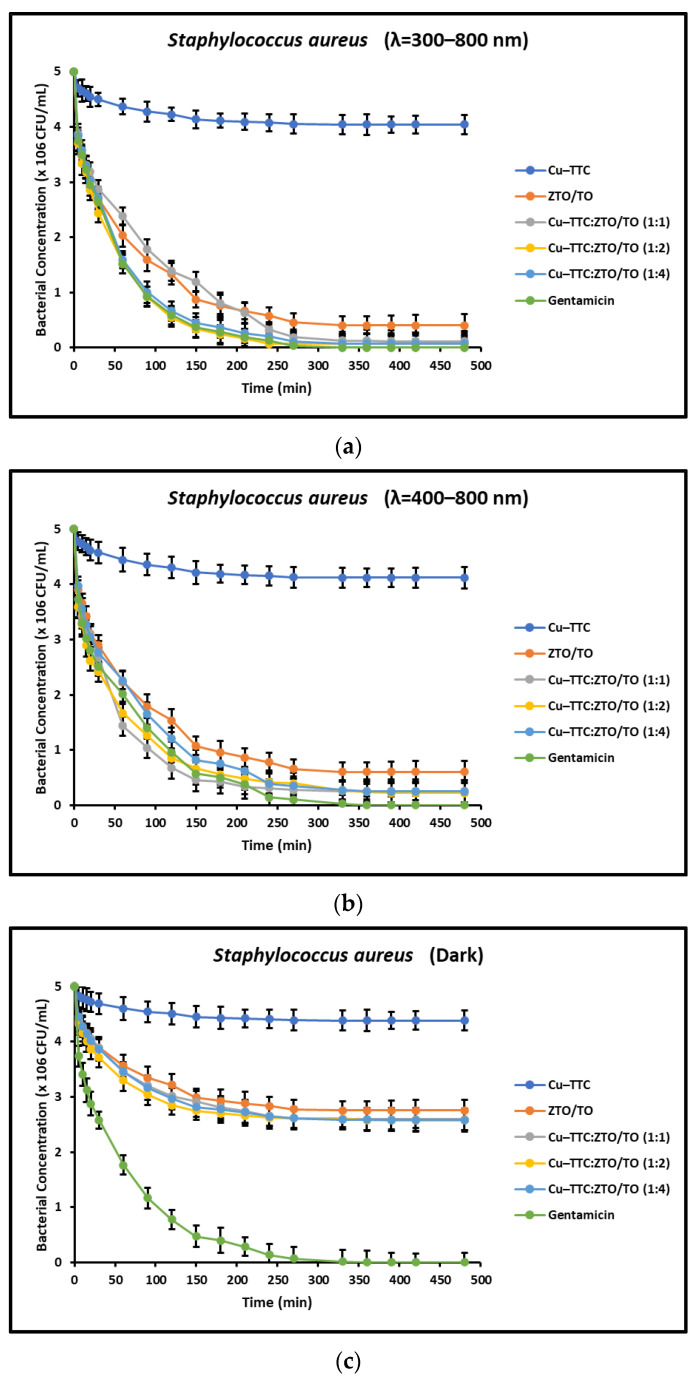
Kinetics of inactivation of *S. aureus* under (**a**) full simulated solar radiation, (**b**) with a 400 nm cut-off filter, and (**c**) in dark conditions.

In this study, the bacterial inactivation dependent on the composition of the catalyst was also evaluated. From the minimal increase in bacterial photoinactivation in the reaction system at concentrations greater than 25 mg/mL, it was decided that this concentration was the optimal operating condition for continuing bacterial photoinactivation experiments. Figure 5a–c show the kinetics of bacterial inactivation for *S. aureus* induced by the coordination complex, the photocatalysts and the antibiotic Gentamicin (1 mg/mL) under different radiation conditions.

**Figure 6:**
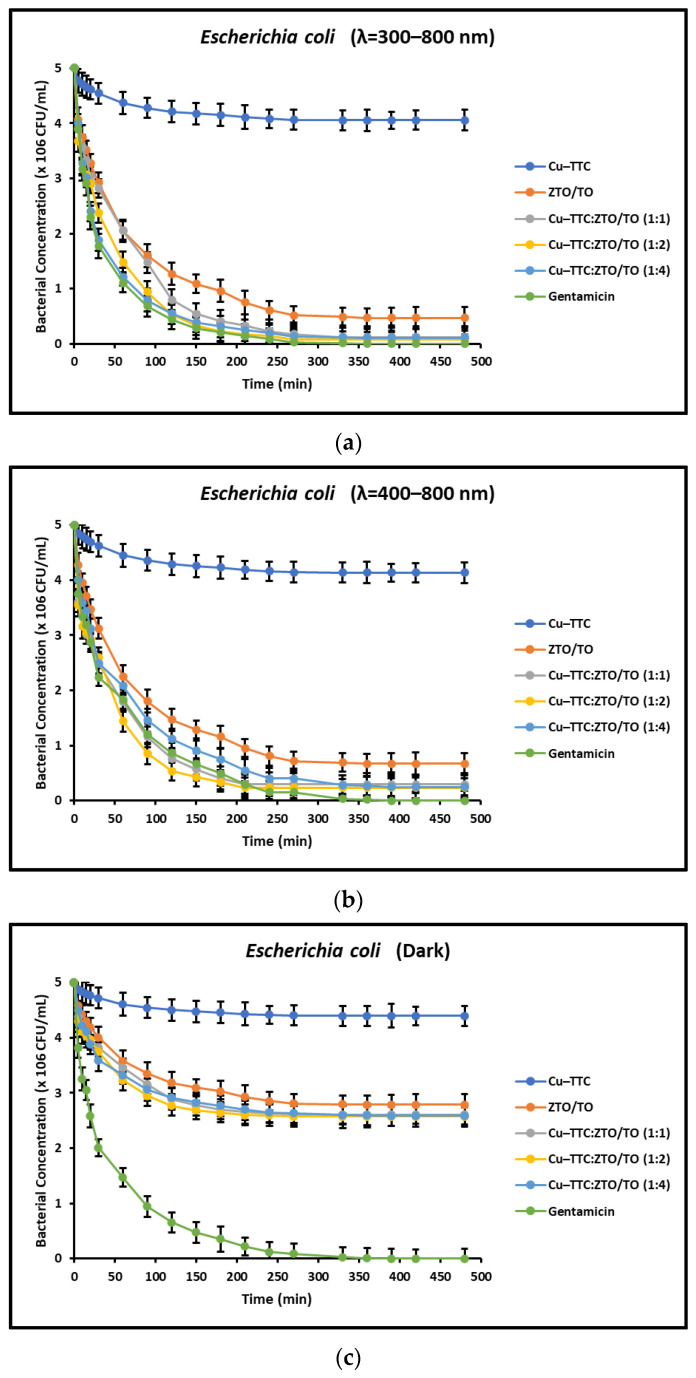
Kinetics of inactivation of *E. coli* under (**a**) full simulated solar radiation, (**b**) with a 400 nm cut-off filter, and (**c**) in dark conditions.

Likewise, Figure 6a–c show the kinetics of bacterial inactivation for *E. coli* induced by the coordination complex, the photocatalysts and the antibiotic Gentamicin (1 mg/mL) under different radiation conditions.

Figure 5a,b and Figure 6a,b present the bacterial inactivation kinetics under simulated solar irradiation. The acceleration effect induced by irradiation evidently leads to complete bacterial inactivation within ~240 min for the ZTO/TO and Cu-TTC/ZTO/TO photocatalysts. In contrast, Figure 5c and Figure 6c show that in the dark, only partial bacterial inactivation occurred within ~240 min for ZTO/TO and Cu-TTC/ZTO/TO due to the lack of contribution of electronic photoexcitation in these photocatalysts. For both *S. aureus* and *E. coli*, the Cu-TTC coordination complex showed little activity, while the antibacterial Gentamicin was effective within ~240 min in all tests.

**Figure 7:**
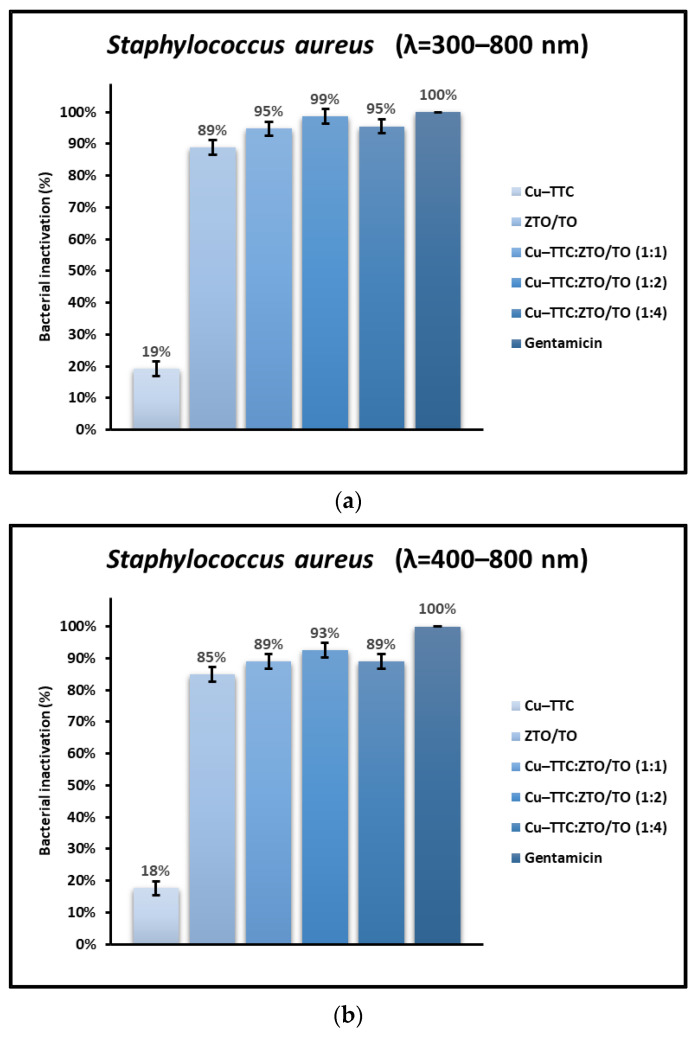

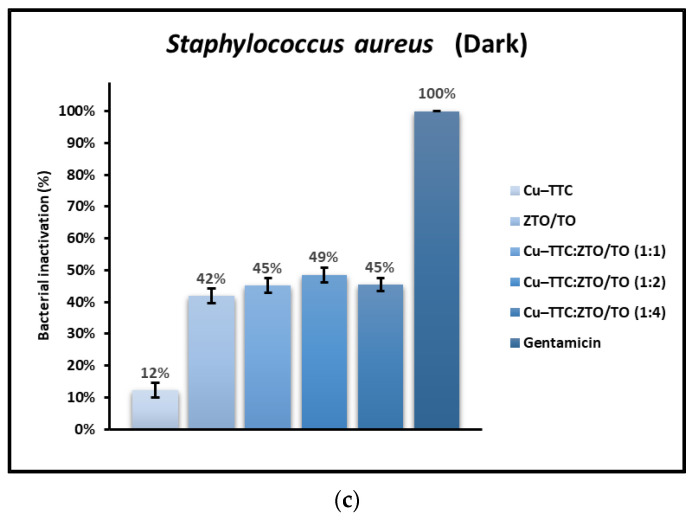
Percentage of inactivation of *S. aureus* under (**a**) total simulated solar radiation, (**b**) with a 400 nm cut-off filter, and (**c**) in dark conditions.

On the other hand, the percentage of dead cells was calculated in relation to the number of initial live cells (CFU/mL) for each bacterium. Figure 7 presents the bacteria photoinactivation (%) for *S. aureus* mediated by the coordination complex, the photocatalysts, and the antibiotic Gentamicin (1 mg/mL) when applying different radiation conditions.

**Figure 8:**
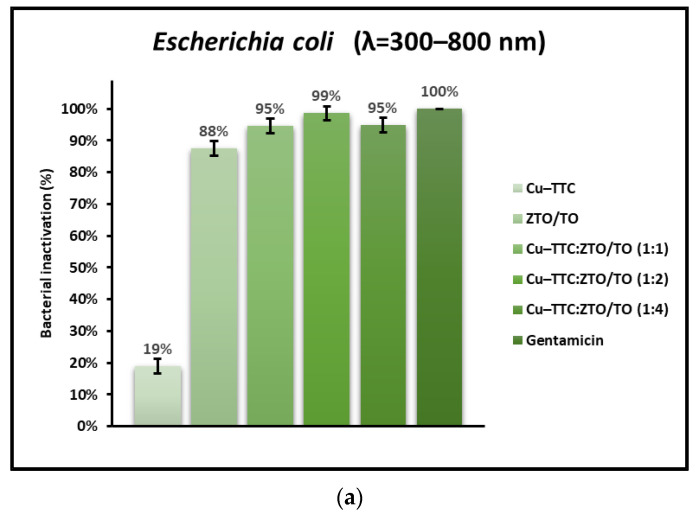

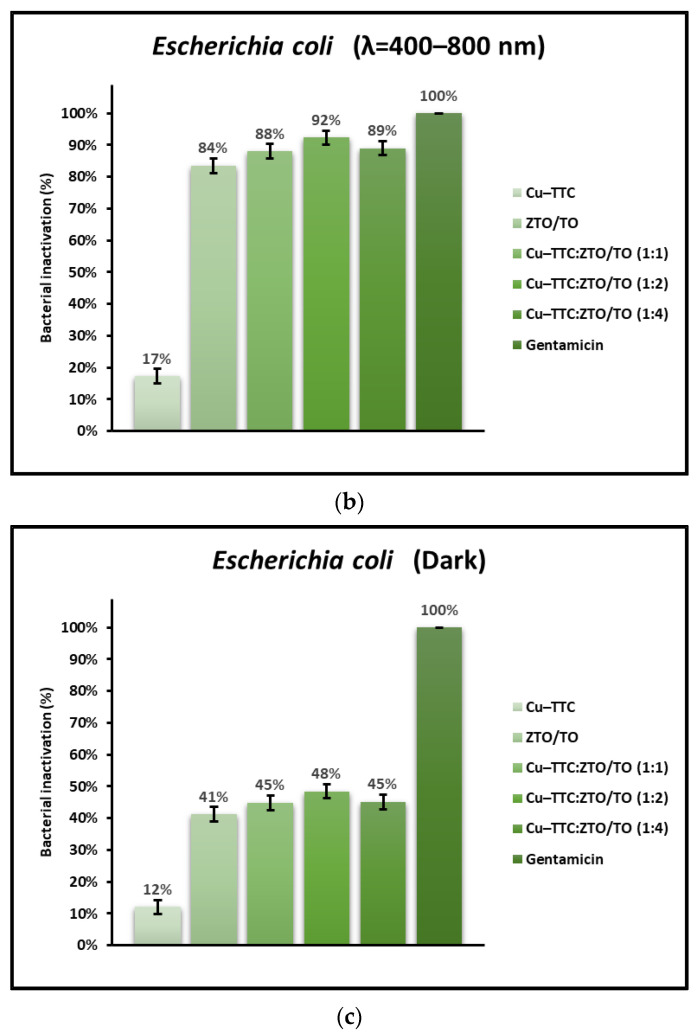
Percentage of inactivation of *E. coli* under (**a**) total simulated solar radiation, (**b**) with a 400 nm cut-off filter, and (**c**) in dark conditions.

Likewise, Figure 8 presents the bacteria inactivation (%) for *E. coli* mediated by the coordination complex, the *photocatalysts*, and the antibiotic Gentamicin (1 mg/mL) when applying different radiation conditions. In this figure, it is seen that the photoinactivation of *E. coli* proceeds with the same trend as that of *S. aureus*.

Figure 7 and Figure 8 show that the percentage of bacterial photoinactivation of Cu-TTC/ZTO/TO increases as the ratio (% *w*/*w*) of the Cu-TTC complex in the ZTO/TO hybrid semiconductor increases from 1:4 to 1:2. However, in the 1:1 ratio (% *w*/*w*), a decrease in antibacterial activity was observed due to the possible high agglomeration of Cu-TTC particles on the surface of the ZTO/TO.

### 3.4. Reuse of Photocatalyst for Bacterial Photoinactivation

**Figure 9:**
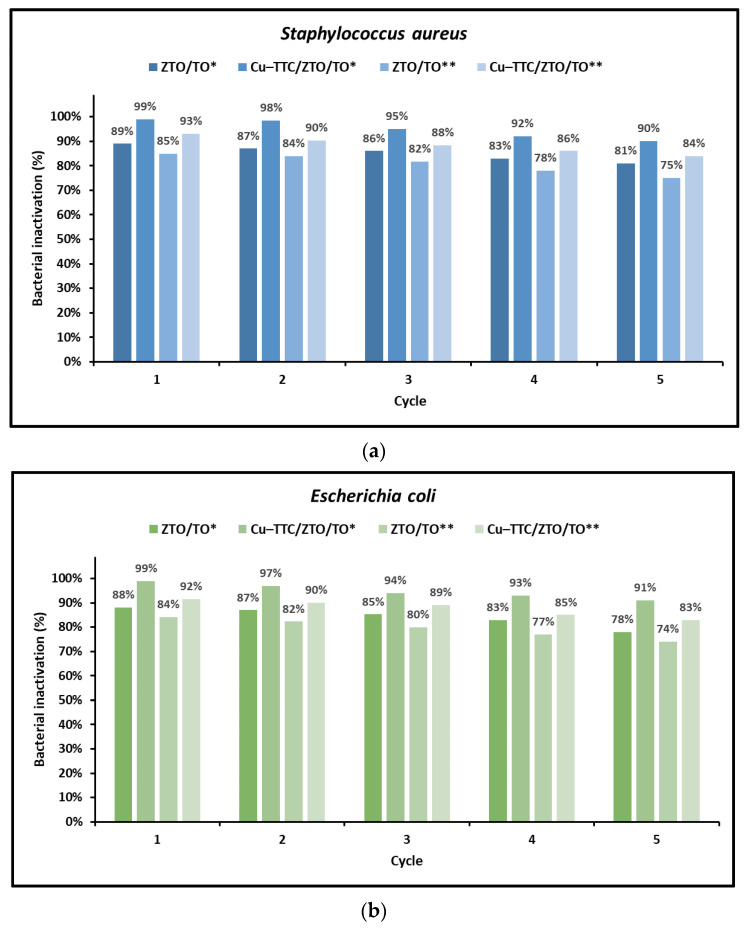
Photoinactivation percentage of (**a**) *S. aureus* and (**b**) *E. coli* under total simulated solar radiation (*) and with a 400 nm cut-off filter (**) for five successive cycles.

Finally, since the recyclability and stability of a photocatalyst are important factors for its large-scale application, five consecutive experiments were carried out in this study to test the reuse of photoinactivators. The results of these assays are shown in Figure 9a,b for *S. aureus* and *E. coli*, respectively.

As shown in Figure 9, for both *S. aureus* and *E. coli*, the results of the reuse experiments showed that, on average, the deactivation of the photocatalysts ZTO/TO and Cu-TTC/ZTO/TO (composition P2) did not exceed the 10% after five consecutive cycles. Evidence from this study suggests that these photocatalysts could have important environmental applications for bacterial photoinactivation in aqueous systems.

## 4. Discussion

### 4.1. Characterization of ZTO/TO and Cu-TTC/ZTO/TO Nanocomposites

As mentioned above, XRD analysis of the composites ZTO/TO and Cu-TTC/ZTO/TO demonstrated the presence of anatase (TiO_2_) and zinc titanate (ZnTiO_3_) in both composites. Likewise, the presence of the coordination complex based on trithiocyanuric acid (TTCA) and Cu(I) was demonstrated in the Cu-TTC/ZTO/TO composite. Several studies reported in the literature have provided sufficient evidence on the antibacterial activity of these compounds. Thus, it is widely known that bacterial cells exposed to anatase (TiO_2_) photocatalysis suffer a series of consequences, including accelerated cell inactivation at the level of regulation and signaling, decreased biosynthesis capacity, degradation of heme groups (Fe-S cluster), decreased capacity to assimilate and transport iron and phosphorus, and decreased coenzyme-independent respiratory chains, among others. These activities, as well as the extensive cell wall alterations, are mainly promoted by the efficient photogeneration of reactive oxygen species (ROS), which is the most important factor supporting the high biocidal performance of the photocatalytic nanomaterials [67,68]. In addition to anatase, other titanium-based photocatalytic materials, including rutile [69], and zinc titanate (ZnTiO_3_) [14], as well as trithiocyanuric acid (TTCA)-based coordination complexes [66] and Cu(I) compounds [4], have shown high efficacy against clinically relevant pathogens. Therefore, it is suggested that Cu-TTC, ZnTiO_3_, and TiO_2_ would be contributing with their individual electrochemical properties (see Table 1) to the overall efficiency of the Cu-TTC/ZTO/TO composite, allowing to obtain a coupled semiconductor, active under solar light, with low recombination capacity of e^−^/h^+^ pairs, high interfacial charge transfer capacity, and a great capacity for ROS generation for effective bacterial photoinactivation.

On the other hand, in this study, the semiconductors ZTO/TO and CU-TTC/ZTO/TO, with particle size <100 nm, reflected a point of zero charge (pH_PZC_) between 7 and 8. Consequently, during the bacterial photoinactivation assays that were performed at a pH lower than pH_PZC_, it is likely that the surface of both photocatalysts could be positively charged, which, according to the literature, would enhance the attraction of the negatively charged microbial cell wall structure, facilitating the bacterial inactivation process [16].

### 4.2. Evaluation of Bacterial Photoinactivation

There are several reports detailing the impact of operating parameters on the bacterial photoinactivation process. These parameters include the pH value, the radiation intensity, as well as the concentration and composition of the photocatalysts in the reaction system. According to the literature, the effect of pH in photocatalytic processes is crucial since the change in the value of this parameter allows modulation of the effective charge of the reaction system. However, there are also several reports in the literature that demonstrate the non-dependence of pH in bacterial disinfection processes. In fact, it has been reported that the bactericidal property in a treatment system can be kept constant between pH 5 and 8 [16].

In this study, the assays were performed without adjusting the pH of the system during the photoinactivation process. The pH measurements before and after the photoreactions showed that the pH decreases (from 6.8 to 5.4) when the Cu-TTC/ZTO/TO or ZTO/TO photocatalysts are irradiated for 8 h using simulated solar light with a 400 nm cut-off filter. However, when the photocatalysts were tested under the full range of simulated solar light, the pH value remained constant (at 6.8) within the same reaction time. According to the literature, the decrease in pH during the reaction with the light of λ > 400 nm could be due to the accumulation of carboxylic acids generated during the peroxidation of the bacterial cell membrane. In contrast, for photocatalysts that react under full simulated solar irradiation (without filter), these carboxylic groups could decompose to CO_2_ due to the presence of UV-A light [70].

Regarding the concentration of the photocatalyst, in this study, it was shown that it is an important operational parameter. In fact, several studies reported in the literature have shown that the bacterial inactivation process improves with increasing photocatalyst concentration until the saturation limit is reached. Figure 3 and Figure 4 display the effect of the concentration of the investigated photocatalysts on the percentage of bacterial inactivation. These figures show that the photocatalysts rapidly reach the saturation limit, after which the inactivation percentage remains constant even with increasing photocatalyst concentration. This is probably due to the fact that the increase in the concentration of the photocatalyst could finally lead to the turbidity of the reaction medium and consequently hinder the absorption of the incoming radiation [16]. Therefore, the experiments carried out in this study allowed us to optimize the concentration of the photocatalyst necessary (25 mg/mL) for the photoinactivation process in order to avoid its excessive use.

Like the concentration of the photocatalyst, the intensity of the irradiation is also an important parameter in the process of bacterial photoinactivation [30]. When the photocatalyst surface is illuminated, the active sites generate reactive oxygen species (ROS) that initiate the process of bacterial photoinactivation. According to the literature, a higher intensity of irradiation can result in a higher rate of ROS production and, consequently, a higher disinfectant effect [16]. In fact, this argument was verified in the present study, since a higher percentage of bacterial photoinactivation was obtained when irradiated with simulated sunlight in the 300–800 nm range compared to irradiation in the 400–800 nm range and in dark conditions.

In addition to the radiation intensity, the electronic properties of the photocatalyst are also an important parameter in the effective rate of ROS production. For a UV-active semiconductor material such as TiO_2_, the rate of bacterial photoinactivation under sunlight is limited, since UV light only makes up a small proportion of the solar spectrum. However, semiconductor coupling and sensitization mediated by metal–organic complexes have proven to be effective methods to improve the quantum efficiency of this oxide and extend its optical adsorption range toward visible light [25,54,71]. The results shown in Figure 5 and Figure 6 suggest that the novel photocatalyst Cu-TTC/ZTO/TO showed a significant improvement in bacterial photoinactivation rate compared to the individual components Cu-TTC and ZTO/TO. This improvement in the photoactivity of the novel semiconductor is possibly due to the fact that the incorporation of the Cu-TTC coordination complex contributes to the efficient separation of the photo-generated e^−^/h^+^ pairs between TiO_2_ and ZnTiO_3_ [58]. Although the incorporation of Cu-TTC improved the effectiveness of the ZnTiO_3_/TiO_2_ hybrid semiconductor, Figure 7 and Figure 8 show that the percentage of bacterial photoinactivation of Cu-TTC/ZTO/TO increases with the proportion of the Cu-TTC complex in the ZTO/TO hybrid semiconductor. However, a slight decrease in antibacterial activity was observed as the proportion of the complex in the hybrid semiconductor continued to increase. These results suggest that the increasing agglomeration of Cu-TTC particles on the surface of the ZTO/TO could have blocked several of its active sites, thus decreasing the photoactivity of this hybrid semiconductor.

Finally, the results shown in Figure 9 suggest the feasibility of using the Cu-TTC/ZTO/TO photocatalyst for several cycles, allowing the effective photoinactivation of *S. aureus* and *E. coli*.

### 4.3. Proposed Mechanism of ROS-Mediated Bacterial Photoinactivation

In photocatalytic processes, both oxidation and reduction reactions take place simultaneously and can generate reactive oxygen species (ROS) such as the superoxide anion radical (^•^O_2_^−^), hydrogen peroxide (H_2_O_2_), singlet oxygen (^1^O_2_), and hydroxyl radical (^•^OH). These reactive species can be detected through various methods, including coloration, chemiluminescence, direct fluorescence, fluorescence probe, direct emissions, direct absorption in UV and IR regions, electron magnetic resonance, and direct electron spin resonance. When photocatalytic processes occur in aqueous systems, ROS can be generated by sequential reactions from both O_2_ and H_2_O. In fact, the stepwise reduction mechanism from O_2_ generates ROS of ^•^O_2_^−^, H_2_O_2_, and ^•^OH, while ROS of ^•^OH, H_2_O_2_, ^•^O_2_^−^, and ^1^O_2_ are generated in this order by the stepwise oxidation mechanism from H_2_O. According to the literature, the surface photocatalytic reactions that are part of these ROS-generating mechanisms can occur at both the anion-bridged OH site and the cationic terminal OH site of semiconductor oxides such as TiO_2_ [68].

Reactive oxygen species (ROS) are essential to initiate bacterial photoinactivation processes. Bacterial photoinactivation under solar light represents a promising antimicrobial strategy, with numerous advantages and diverse potential applications [72,73,74,75,76]. According to the literature, photoexcited catalysts can inactivate various types of microorganisms as a result of the movement of photogenerated electron–hole (e^−^/h^+^) pairs on the catalyst surface. The electrons moving toward the catalyst surface react with O_2_ in solution to generate ^•^O_2_ and ^•^OH radicals. These radicals can oxidize the cell membrane of microorganisms, achieving their photoinactivation [77].

As reported in the literature, ROS-mediated bacterial inactivation has benefits over other antimicrobial treatments including the use of antibiotics and exposure to ultraviolet light [78]. This is probably due to the fact that the ROS generated can cause non-specific damage (they do not have specific cellular target molecules) preventing organisms from developing genetic mutations and acquiring resistance. Furthermore, nonspecific oxidative damage to exposed microorganisms facilitates the efficient inactivation of a wide range of microbial species [79].

In this study, the results confirm that photoinactivation of *S. aureus* and *E. coli* bacteria is improved when Cu-TTC/ZTO/TO is used instead of ZTO/TO photocatalyst or Cu-TTC complex alone. This agrees with the results obtained in a previous study indicating that the Cu-TTC/ZTO/TO photocatalyst has higher photoactivity than the ZTO/TO hybrid semiconductor [58,80] due to the fact that the Cu-TTC complex could contribute to the efficient separation of photogenerated e^−^/h^+^ pairs between ZnTiO_3_ and TiO_2_ [81]. Although the optical absorption properties of ZTO/TO, Cu-TTC and Cu-TTC/ZTO/TO were not determined here, Table 1 shows the corresponding values of the maximum absorption peaks, the molar extinction coefficients (ε) and the bandgap energy (E_g_) which were calculated from the previously obtained electronic absorption UV–vis spectra. From these results and based on the electronic properties of the ZTO/TO and Cu-TTC/ZTO/TO semiconductors and the geometry of the previously reported Cu-TTC complex [57,82], it was suggested that the strong absorption band of the Cu-TTC/ZTO/TO photocatalyst Cu-TTC/ZTO/TO (745 nm) in the visible region, compared to the absorption band of the ZTO/TO photocatalyst (390 nm), is due to the state of metal–ligand charge transfer (MLCT), as well as to the π **→** π* transitions in triazine rings of the Cu-TTC complex. Therefore, when Cu-TTC/ZTO/TO is irradiated by simulated solar light, the electrons in the Cu-TTC complex can be easily excited from the ground state probably because of the MLCT excitations and π **→** π* transitions to then move freely on the surface of the ZTO/TO hybrid semiconductor, improving its photoelectrochemical performance under solar light. Table 1 also shows the effect of the Cu-TTC complex in the reduction of the bandgap energy of the ZnTiO_3_/TiO_2_ hybrid semiconductor. Therefore, the Cu-TTC/ZTO/TO composite (E_g_ = 2.54 eV) could have higher photochemical efficiency than the ZTO/TO semiconductor (E_g_ = 3.06 eV), constituting a promising alternative for bacterial photoinactivation under solar light.

**Figure 10:**
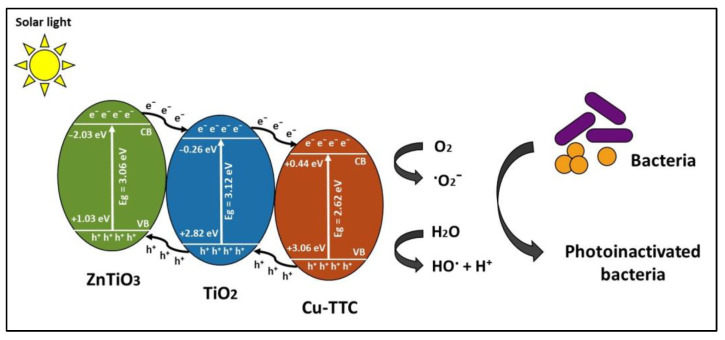
Bacterial photoinactivation mechanism.

Figure 10 shows the proposed mechanism for bacterial photoinactivation mediated by Cu-TTC/ZTO/TO composite. The values of the valence band (VB) and conduction band (CB) potentials of Cu-TTC (+0.44, +3.06 eV), TO (−0.26, +2.86 eV), and ZTO (−2.03, +1.03 eV) shown in the figure were estimated in a previous study using Mulliken’s theory of electronegativity [58]. From this figure, it is suggested that bacterial inactivation could involve interfacial charge transfer (IFCT) between the surfaces of the hybrid semiconductor ZTO/TO and the Cu-TTC coordination complex. In this figure, it is evident that the excited electrons from the conduction band (CB) of ZnTiO_3_ are transferred to the CB of TiO_2_ and then to the CB of Cu-TTC, while the highly oxidative holes generated by the valence band (VB) of Cu-TTC are transferred to the VB of TiO_2_ and then to the VB of ZnTiO_3_, resulting in a suitable energy cascade. This multielectron process improves the mobility of electrons in the compound and reduces interfacial electron/pair recombination, thus promoting an enhanced photochemical effect [83,84].

According to the literature, photogenerated holes in photocatalysts involve oxygen-trapped sites in the lattice. Therefore, electrons can reduce Ti^4+^ to Ti^3+^, creating oxygen vacancies in the photocatalyst lattice. Oxygen vacancies increase the affinity for water as the surface becomes more hydrophilic [48]. Consequently, it is suggested that *Staphylococcus aureus* (Gram (+)) and *E. coli* (Gram (−)) bacteria will present a greater adhesion to the hydrophilic surface of the ZTO/TO hybrid photocatalyst, facilitating their subsequent photoinactivation.

Furthermore, Figure 10 shows that the Cu-TTC/ZTO/TO semiconductor may offer an alternative route to generate reactive oxygen species (ROS), which are critical in many photochemical processes. As mentioned before, bandgap excitation under simulated sunlight radiation generated conduction band electrons and valence band holes in the semiconductor. In the following mechanism, it is proposed that the activation of molecular O_2_ could proceed by accepting electrons from the conduction band of the semiconductor, generating O_2_˙ˉ. The production of H_2_O_2_, by disproportionation of O_2_˙ˉ after protonation (R1 and R2 reactions) is possible through a Fenton-like mechanism to provide ·OH radicals (R3 and R4 reactions) [85]. Such a mechanism implies that Cu-TTC/ZTO/TO acts as a catalyst to ultimately produce the highly reactive species during photocatalysis. These reactive oxygen species (ROS), in particular ·OH (R3 and R4 reactions), are essential for bacterial photoinactivation (R5 reaction).

O_2_*˙ˉ* + H^+^ → HO_2_˙, (R1)

2HO_2_˙ → O_2_ + H_2_O_2_, (R2)

Cu^+^ + H_2_O_2_ → Cu^2+^ + ·OH + OH^−^, (R3)

H_2_O_2_ + O_2_˙ˉ → ·OH + OH^−^ + O_2_, (R4)

·OH or h^+^ + bacteria → photoinactivation. (R5)

In our early reports, we demonstrated the presence of the Cu^+^ oxidation state with some Cu^2+^ impurity in the Cu-TTC photoactive coordination complex [57]. Therefore, the conduction band electron of the complex is also capable of activating molecular oxygen, generating free or coordinated superoxo species, Cu^2+^-O_2_˙ˉ, and then H_2_O_2_ (R1 and R2 reactions). In addition, according to the literature, Cu^+^ can significantly generate ·OH (R3 reaction), although it should be noted that bandgap excitation and R3 reaction generate Cu^2+^ species. However, the recovery of Cu^+^ in Cu-TTC could be possible due to the combination with the semiconductors ZnTiO_3_ and TiO_2_, with which it forms a type II heterojunction [85].

## 5. Conclusions

This study demonstrated the effect of the type of radiation and the concentration/composition of the photocatalyst on the photoinactivation process of *S. aureus* and *E. coli* bacteria. Greater bacterial photoinactivation was observed in the light range of 300–400 nm, using 25 mg/mL of photocatalyst Cu(C_3_H_3_N_3_S_3_)_3_/ZnTiO_3_/TiO_2_ (Cu-TTC/ZTO/TO) synthesized with a ratio (*w*/*w*%) 1:2 Cu-TTC:ZTO/TO. The faster bacterial inactivation observed for the Cu-TTC/ZTO/TO samples relative to the ZTO/TO and Cu-TTC samples is probably due to interfacial charge transfer (IFCT) between the ZTO/TO and Cu-TTC surfaces, which leads to faster bacterial inactivation with respect to ZTO/TO and Cu-TTC samples.

The results of this study suggest that light irradiation induces h^+^ in the ZTO/TO valence band (VB) with high oxidative power. Electrons transferred from ZTO/TO to Cu-TTC reduce oxygen. The oxygen reduction would proceed through a multi-electronic process. Holes in the VB of ZTO/TO lead to bacterial inactivation. At the same time, Cu(I), due to interfacial charge transfer (IFCT), reduces adsorbed oxygen and can act catalytically under visible light, which also leads to bacterial inactivation. Therefore, it is suggested that the Cu-TTC/ZTO/TO compound could offer an effective route to generate reactive oxygen species (ROS), which are essential for bacterial photoinactivation.

## Figures and Tables

**Table 1:** Maximum absorption peaks, molar extinction coefficients (ε), and bandgap energy (E_g_) for ZTO/TO, Cu-TTC, and Cu-TTC/ZTO/TO.

Material	Absorbance (a.u.)	Wavelength (λ_max_) (nm)	Molar Extinction Coefficient (ε) (10^4^ M^−1^ cm^−1^)	Band Gap Energy (E_g_) (eV)
ZTO/TO	0.48	390	1.92	3.06
Cu-TTC	0.60	725	2.40	2.62
Cu-TTC/ZTO/TO	0.69	745	2.76	2.54

## Data Availability

Data are available from the authors upon reasonable request.

## Supplementary Materials

The following supporting information can be downloaded at: https://www.mdpi.com/article/10.3390/nano13010173/s1. Figure S1. Tautomeric structures of trithiocyanuric acid (TTCA); Figure S2. Anchoring modes of the tautomer-thione Cu-TTC molecule on the surface of (a) ZnTiO_3_ and (b) TiO_2_.
