# Supplementary material for: Novel Semiconductor Cu(C3H3N3S3)3/ZnTiO3/TiO2 for the Photoinactivation of E. coli and S. aureus under Solar Light
Source: Nanomaterials (Basel). 2022 Dec 30;13(1):173. doi: 10.3390/nano13010173 (PMC9824406; doi:10.3390/nano13010173)
Supplement: Supplementary file 1 [file nanomaterials-13-00173-s001.zip › nanomaterials-2112114-supplementary.pdf]

nanomaterials

*Supplementary Material*

# Novel semiconductor $Cu(C_3H_3N_3S_3)_3/ZnTiO_3/TiO_2$ for the Photoinactivation of *E. coli* and *S. aureus* under Solar Light

**Ximena Jaramillo-Fierro [1,*], María Fernanda Cuenca [2]**

1 Departamento de Química, Facultad de Ciencias Exactas y Naturales, Universidad Técnica Particular de Loja, San Cayetano Alto, Loja 1101608, Ecuador
2 Departamento de Producción, Facultad de Ciencias Exactas y Naturales, Universidad Técnica Particular de Loja, San Cayetano Alto, Loja 1101608, Ecuador; mfcuenca70@utpl.edu.ec
* Correspondence: xvjaramillo@utpl.edu.ec; Tel.: +593-7-3701444

**Abstract:** The use of semiconductors for bacterial photoinactivation is a promising approach that has attracted great interest in wastewater remediation. The photoinactivator Cu-TTC/ZTO/TO was synthesized by the solvothermal method from the coordination complex $Cu(C_3H_3N_3S_3)_3$ (Cu-TTC) and the hybrid semiconductor $ZnTiO_3/TiO_2$ (ZTO/TO). In this study, the effect of photocatalyst composition/concentration as well as radiation intensity on the photoinactivation of the gram-negative bacteria *Escherichia coli* and the gram-positive bacteria *Staphylococcus aureus* in aqueous solutions was investigated. The results revealed that 25 mg/mL of photoinactivator, in a Cu-TTC:ZTO/TO molar ratio of 1:2 (w/w%), presents a higher rate of bacterial photoinactivation under simulated solar light ($\lambda$ = 300-800 nm), in comparison to the individual components. The evidence of this study suggests that the presence of the $Cu(C_3H_3N_3S_3)_3$ coordination complex in the $ZnTiO_3/TiO_2$ hybrid semiconductor would contribute to the generation of reactive oxygen species (ROS) that are essential to initiate the bacterial photoinactivation process. Finally, the results obtained allow us to predict that the Cu-TTC/ZTO/TO photocatalyst could be used for effective bacterial inactivation of *E. coli* and *S. aureus* in aqueous systems under simulated solar light.

**Keywords:** Semiconductors; reactive oxygen species (ROS); bacterial photoinactivation; *Staphylococcus aureus*; *Escherichia coli*; solar light.

**Citation:** Jaramillo-Fierro, X.; Cuenca, M.F. Novel Semiconductor $Cu(C_3H_3N_3S_3)_3/ZnTiO_3/TiO_2$ for the Photoinactivation of *E. coli* and *S. aureus* under Solar Light. *Nanomaterials* **2023**, *13*, x. https://doi.org/10.3390/nano13010173

Academic Editor: Vincenzo Vaiano

Received: 12 December 2022
Revised: 23 December 2022
Accepted: 27 December 2022
Published: 30 December 2022

**Copyright:** © 2022 by the authors. Submitted for possible open access publication under the terms and conditions of the Creative Commons Attribution (CC BY) license (https://creativecommons.org/licenses/by/4.0/).

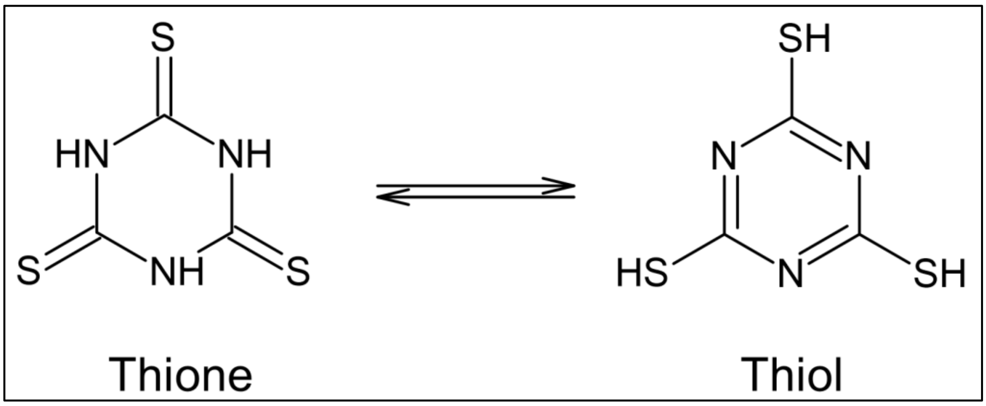


**Figure S1.** Tautomeric structures of trithiocyanuric acid (TTCA).

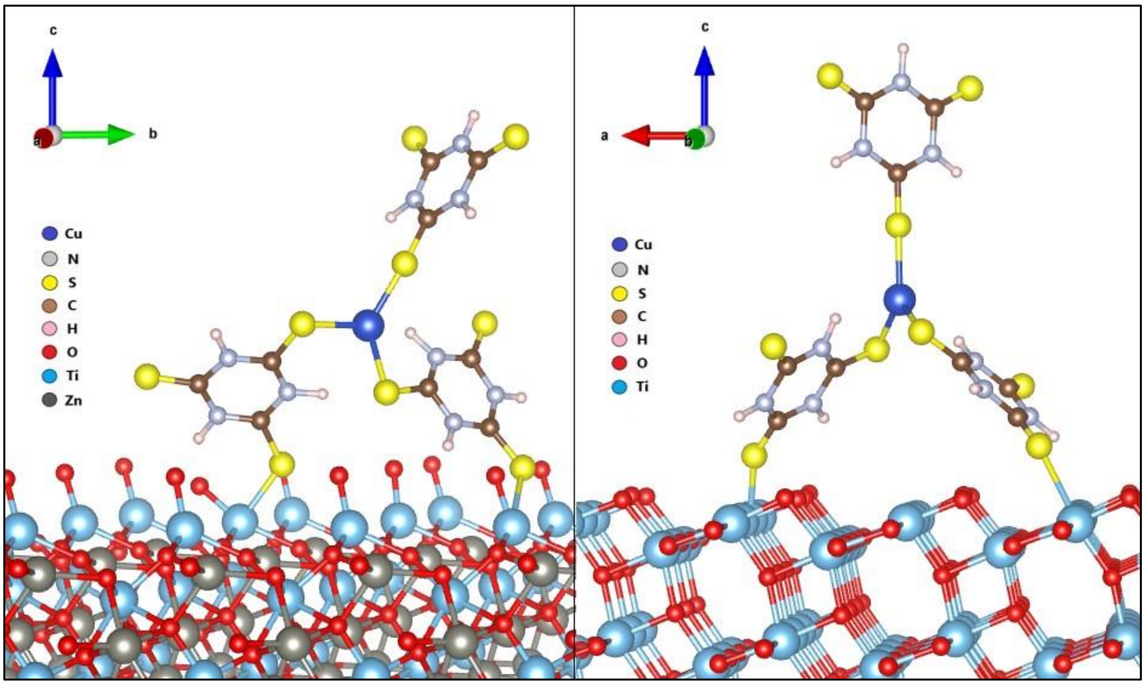


(**a**) (**b**)

**Figure S2.** Anchoring modes of the tautomer-thione Cu-TTC on the surface of (**a**) $ZnTiO_3$ and (**b**) $TiO_2$.
